# The hyaluronan-mediated motility receptor RHAMM promotes growth, invasiveness and dissemination of colorectal cancer

**DOI:** 10.18632/oncotarget.19904

**Published:** 2017-08-03

**Authors:** Valentina Mele, Lena Sokol, Viktor Hendrik Kölzer, Dennis Pfaff, Manuele Giuseppe Muraro, Irene Keller, Zahnd Stefan, Irene Centeno, Luigi Maria Terracciano, Heather Dawson, Inti Zlobec, Giandomenica Iezzi, Alessandro Lugli

**Affiliations:** ^1^ Cancer Immunotherapy, Department of Biomedicine, University of Basel, Basel, Switzerland; ^2^ Translational Research Unit, Institute of Pathology, University of Bern, Bern, Switzerland; ^3^ Cantonal Hospital Baselland, Institute of Pathology, Liestal, Switzerland; ^4^ Cell Signaling, Department of Biomedicine, University of Basel, Basel, Switzerland; ^5^ Oncology Surgery and Department of Biomedicine, University Hospital Basel and University of Basel, Basel, Switzerland; ^6^ Department of Clinical Research and Swiss Institute of Bioinformatics, University of Bern, Bern, Switzerland; ^7^ Molecular Pathology Division, Institute of Pathology, University Hospital, Basel, Switzerland; ^8^ Clinical Pathology Division, Institute of Pathology, University of Bern, Bern, Switzerland; ^9^ Clinical Pathology, Institute of Pathology, University Hospital, Basel, Switzerland

**Keywords:** RHAMM, colorectal cancer, metastasis, invasion, proliferation

## Abstract

In colorectal cancer (CRC), RHAMM is an independent adverse prognostic factor. The aim of the study was therefore to investigate on the role of RHAMM as a potential direct driver of cell proliferation and migration in CRC cell lines and to identify pathways dependent on RHAMM in human CRC.

Proliferation, cell cycle alterations and invasive capacity were tested in two RHAMM- and control- knockdown CRC cell lines by flow cytometry and *in vitro* assays. Tumorigenicity and metastasis formation was assessed in immunodeficient mice. RNA-Seq and immunohistochemistry was performed on six RHAMM+/- primary CRC tumors.

*In vitro*, silencing of RHAMM inhibited CRC cell migration and invasion by 50% (p<0.01). *In vivo*, RHAMM knockdown resulted in slower growth, lower tumor size (p<0.001) and inhibition of metastasis (p<0.001). Patients with RHAMM-high CRC had a worse prognosis (p=0.040) and upregulated pathways for cell cycle progression and adhesion turnover.

RHAMM overexpression is correlated with increased migration and invasion of CRC cells, leads to larger, fast growing tumors, and its downregulation essentially abolishes metastasis in mouse models. RHAMM is therefore a promising therapeutic target in all CRC stages as its inhibition affects growth and dissemination of the primary CRC as well as the metastases.

## INTRODUCTION

Colorectal cancer (CRC) is one of the leading causes of cancer-related deaths in western countries. Over a third of the patients diagnosed with CRC will die within 5 years, mostly from widespread metastatic disease [1, 2]. Targeted therapy approaches are currently only being used to treat patients with metastatic CRC, outlining the need to identify additional therapeutic targets which may help to prevent tumor dissemination.

RHAMM (Receptor for Hyaluronic Acid Mediated Motility/HMMR/CD168) was first characterized as a protein involved in cell locomotion. Today, RHAMM is known to be a versatile protein, typically activated in wound healing [3], and found on the cell surface, in the cytoplasm, and in the nucleus of many different cell types [4]. In response to hyaluronic acid (HA), TGF-β or PDGF [5], RHAMM can activate various signaling cascades. For instance, by binding to proteins such as ERK1/2 [6, 7], FAK [8], or Src [9], RHAMM was reported to activate the respective pathways. Additionally, RHAMM can target MEK1 and ERK1/2 to the cytoskeleton and nucleus, thus regulating mitotic spindle integrity, cell cycle progression, and expression of genes governing reorganization and degradation of the extracellular matrix (ECM). It is functionally linked to key centrosomal proteins such as the Aurora kinase A [10–12], and is essential for bipolar spindle formation and cell division fidelity [13, 14].

Overexpression of RHAMM has been linked to disease progression in many solid [15, 16] and haematological cancers [17]. We have previously described RHAMM as an independent adverse prognostic factor in mismatch repair (MMR) proficient tumors in a cohort of 1420 CRC patients [18]. Further analyses confirmed that the prognostic impact of RHAMM ranked above other established prognostic factors, such as tumor grade and vascular invasion [19]. Additionally, in a recent study, we have analyzed RHAMM expression in highly aggressive tumor cells, termed tumor buds [20]. These are defined as clusters of up to 4 cells at the invasive front and thought to be detached, motile tumor cells in the process of epithelial-mesenchymal-like transition and metastatic dissemination. A high number of buds at the invasion front is one the strongest adverse prognostic factor in CRC [21]. We speculated that RHAMM overexpression on tumor buds would increase motility, invasion into lymph and blood vessels and dissemination, thus contributing to an adverse outcome. Indeed, the presence of RHAMM-positive tumor buds correlated strongly with aggressive clinicopathological features and added significant prognostic information to the tumor-node-metastasis (TNM) classification [20].

Therefore, based on our previous studies, and the literature describing the many key processes this protein is involved in, RHAMM can be described as a very promising prognostic factor and a potential therapeutic target in CRC. Previous molecular signaling pathway studies indicate that RHAMM could be a direct driver of cell proliferation and migration, which might contribute to early metastatic dissemination, which has, to our knowledge, never been tested in CRC. In the present study, we analyzed the effect of RHAMM expression on the proliferation, cell cycle, migration and invasive potential of CRC cell lines and the growth and tumorigenicity of CRC tumors *in vivo*. We additionally use RNA sequencing to identify specific transcripts and pathways which are characteristic for patients with RHAMM positive or negative CRC tumors.

## RESULTS

### Patient survival is correlated to RHAMM expression in tumor cells

First, we re-tested the ability of RHAMM expression to predict survival in a new, well characterized colorectal cancer cohort. Patients with RHAMM high tumors had a significantly lower survival rate (p=0.040, HR 1.50, 95% CI 1.02-2.21, Supplementary Figure 1).

### Subcellular distribution of RHAMM in CRC cell lines

RHAMM levels in a panel of eight established human CRC cell lines (LoVo, DLD-1, HCT116, HCT15, SW620, SW480, HT29, and CACO-2, Supplementary Figure 2A,) were analyzed by flow cytometry. High levels of cytoplasmic RHAMM were detected in all tested cell lines (Supplementary Figure 2B).

Based on our previous studies, we selected a MMR proficient (MMR+, HT29) and a deficient (MMR-, HCT116) cell line for subsequent functional assays. To test for cell surface localization of RHAMM, we labeled both cell lines with an anti-RHAMM antibody. While the levels of intracellular RHAMM were fairly constant, surface RHAMM expression was transient after plating, and could be detected on a small fraction of cells 48 hours after plating, at 50% confluency (data not shown). We confirmed previous reports that RHAMM surface expression could be induced and increased up to fourfold under the same culture conditions by the addition of 100 μg/ml of its extracellular matrix ligand, HA, to the medium (single experiment, Supplementary Figure 3).

### Inhibition of RHAMM slows proliferation and impacts cell cycle progression of HT29 and HCT116 cells *in vitro*

**Figure 1:**
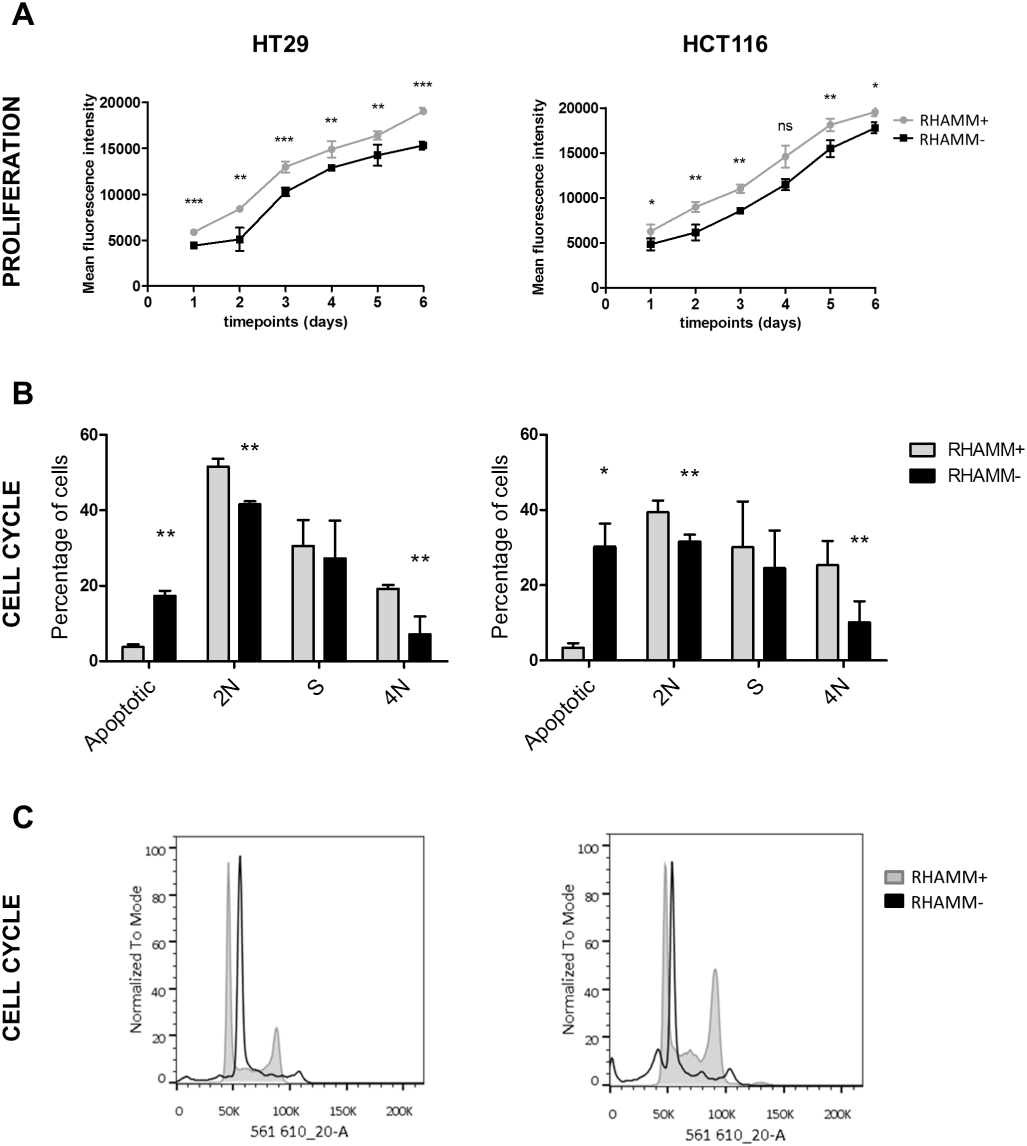
RHAMM silencing reduces the rate of proliferation of tumor cells **(A)** RHAMM+ and RHAMM- HT29 and HCT116 were seeded in a 96-well plate and the proliferation was analyzed every 24 hours for 6 days. The data is plotted as mean +/- standard deviation (SD). **(B, C)** RHAMM silencing reduces the occupancy of the S and G2/M phase of the cell cycle. HT29 and HCT116 cells were fixed, stained with PI/RNase staining buffer (BD), and analyzed on the BD LSRFortessa. The data are an average of three independent experiments.

As levels of RHAMM were high all in CRC cells, we did not additionally test RHAMM overexpression. HT29 and HCT116, which were stably transduced with lentiviral particles carrying either a RHAMM shRNA (RHAMM-) or a scrambled control (RHAMM+, Supplementary Figure 4) were analyzed by CYQUANT cell proliferation assay and flow cytometry for differences in the proliferation rate and cell cycle. RHAMM silencing had a mild, but statistically significant effect on the cell line’s growth, reducing the proliferation of HT29 and HCT116 cells and simultaneously lowering the percentage of cells in the S phase (Figure 1).

### Inhibition of RHAMM decreases motility and invasiveness of HT29 and HCT116 cells *in vitro*

**Figure 2:**
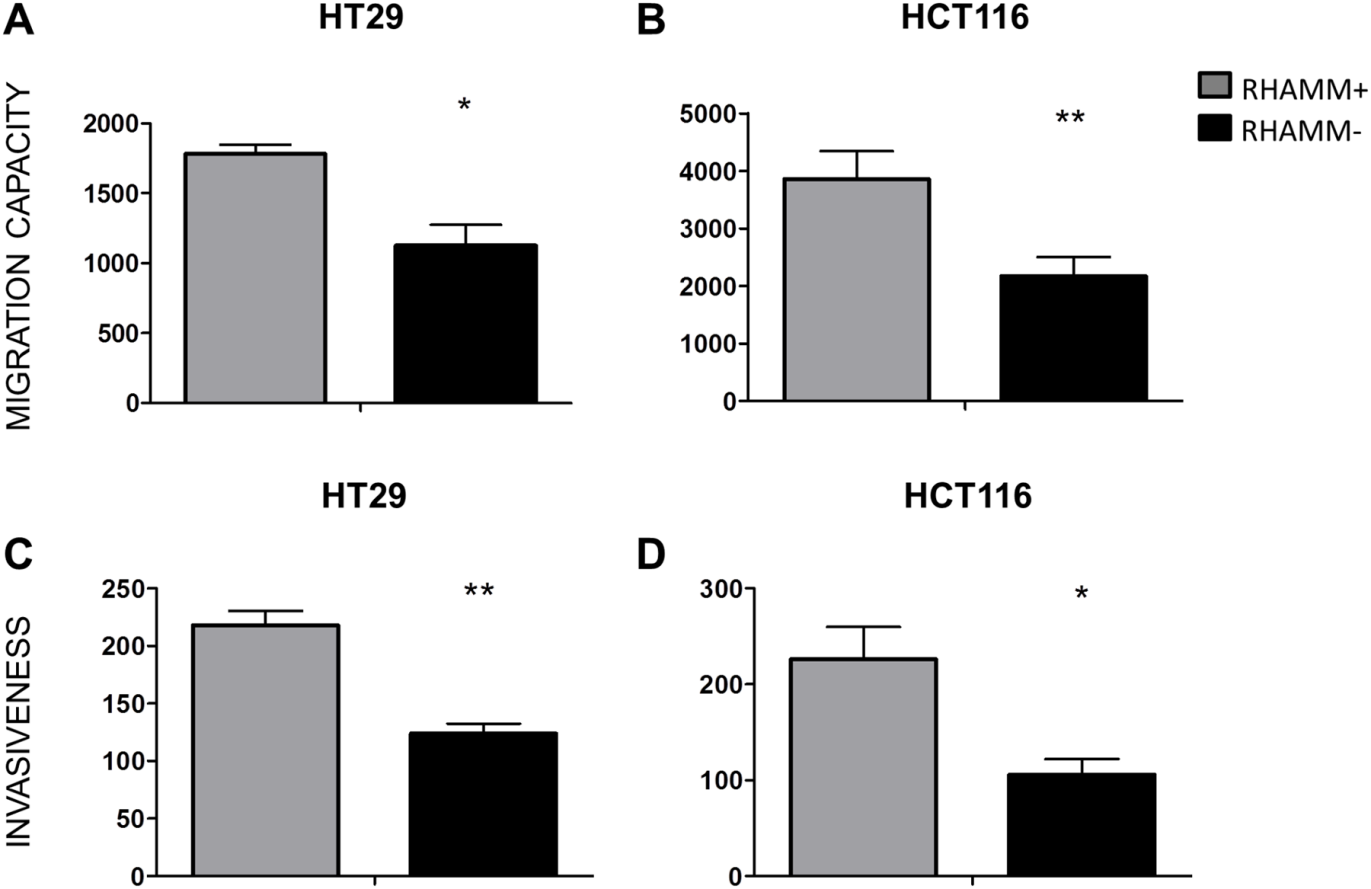
RHAMM silencing reduces the ability of CRC cells to migrate and invade *in vitro* Migration and invasion of HT29 **(A, C)** and HCT116 **(B, D)** tumor cells. The cells were seeded in the upper chambers of transwell plates onto uncoated (A, B) or Matrigel-coated (C, D) membranes (8 μm pores, Corning). At T=20 hours, the number of cells that had migrated into the lower chambers were quantified by CyQUANT Cell Proliferation Assay Kit® (Invitrogen). *p≤0.05, **p≤0.01, Students’ t-test (average from 3 independent experiments). The data is shown as mean +/- SD.

To test the effect of RHAMM on cell motility, the migration of RHAMM- and RHAMM+ HT29 and HCT116 cells was tested in a transwell assay. The amount of cells that migrated through both uncoated membranes and those coated with a Matrigel extracellular matrix was significantly higher in RHAMM+ than in RHAMM- cells (50% increase on average, p<0.01, Figure 2).

### RHAMM positive tumors are more tumorigenic *in vivo*

**Figure 3:**
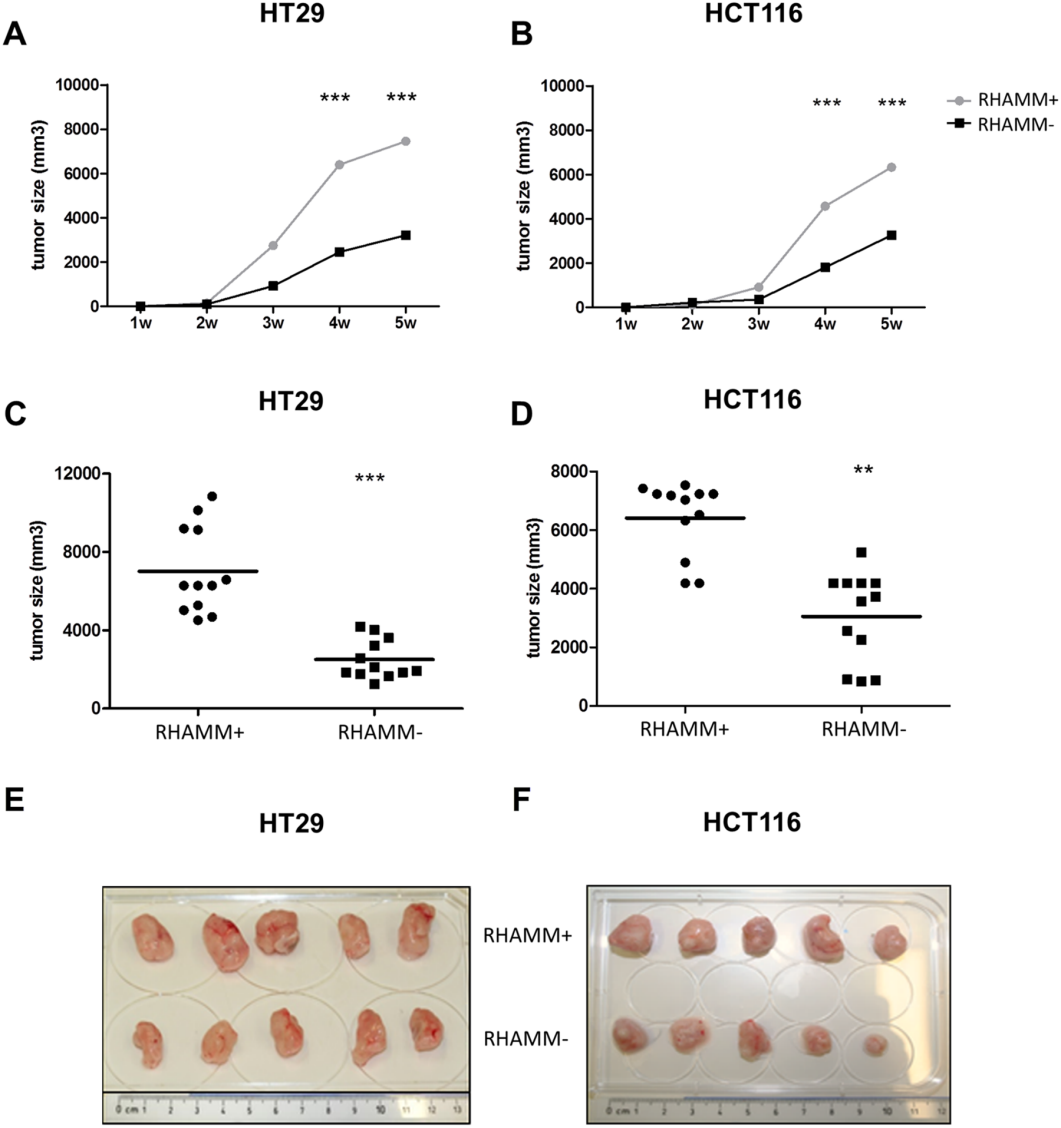
RHAMM silencing reduces the tumorigenicity of CRC cells *in vivo* Immunodeficient NGS mice (n=4 per group) were injected subcutaneously with 10^5^ control or RHAMM- HT29 or HCT116 cell. **(A, B)** Growth kinetics of control and RHAMM- HT29 and HCT116 cells are shown. The data is and plotted as mean +/- SD. **(C, D)** Final tumor volume of HT29 and HCT116 tumors, with or without RHAMM expression, 5 weeks after injection. Data from three separate experiments were pooled. **p≤0.01, ***p≤0.001, Students’ t-test. **(E, F)** HT29 and HCT116 tumors.

RHAMM- and RHAMM+ HT29 and HCT116 cells were injected subcutaneously into immunodeficient NSG mice. Tumor growth was followed for up to 5 weeks. Already at three (HT29) or four (HCT116) weeks post-injection, a significant difference in tumor sizes could be seen. The final average size of RHAMM+ tumors was twofold higher than the RHAMM- tumors (Figure 3, 7500 mm^3^ versus 2800 mm^3^, respectively for HT29 p<0.01, and 6200 mm^3^ versus 3100 mm^3^, respectively for HCT116 tumors p<0.001).

**Figure 4:**
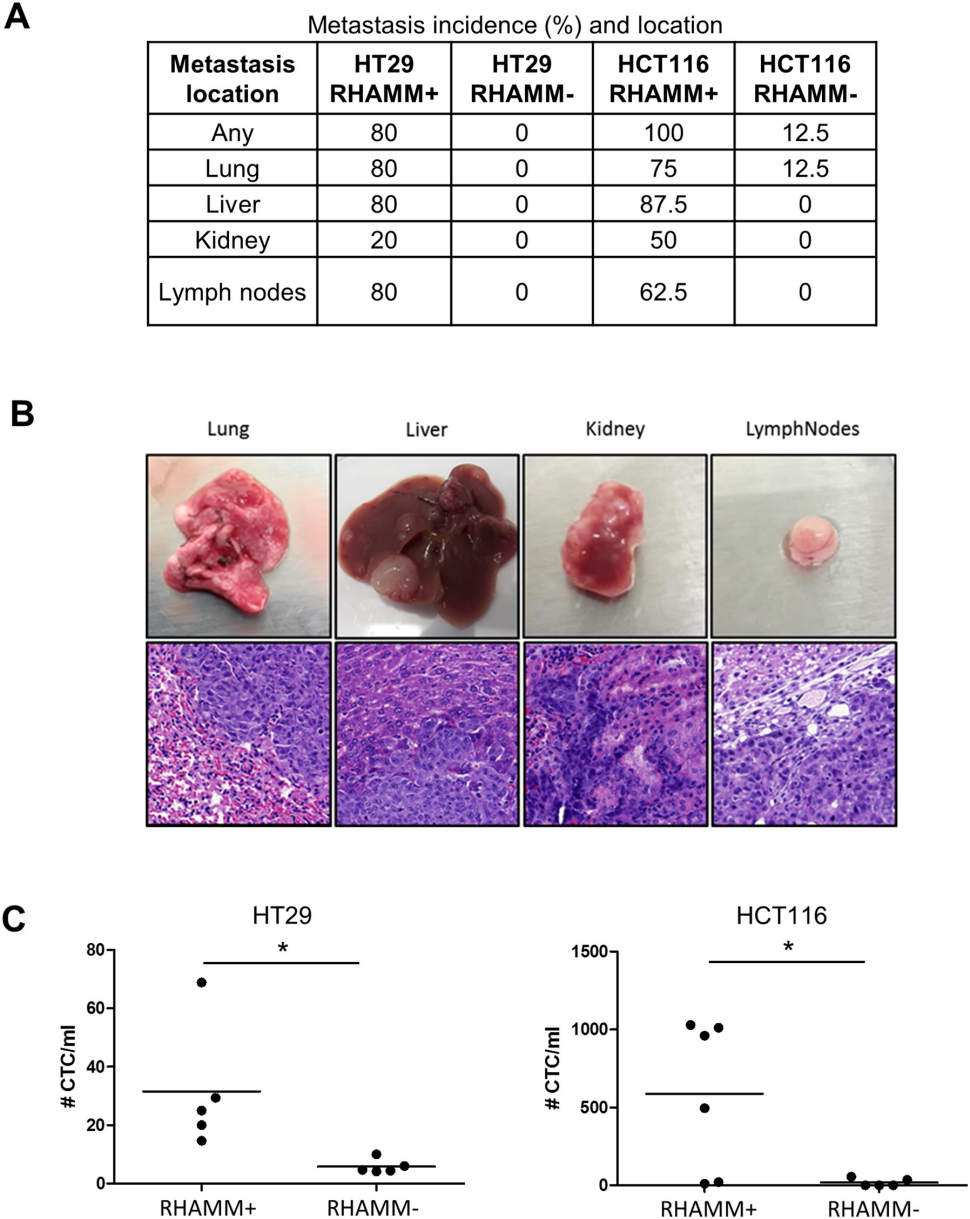
RHAMM silencing suppresses the formation of metastases upon intravenous injection of HCT116 or HT29 cells **(A)** Incidence and location of metastases at the 4 week endpoint. **(B)** Tissues (lung, liver, kidney and lymph nodes) and corresponding hematoxylin and eosin stains of representative metastases induced by HCT116 wild type cells. **(C)** Number of circulating tumor cells (CTC) detected by EpCAM positivity normalized to milliliter of peripheral blood.

To test the effect of RHAMM silencing on the metastatic potential of colon cancer cells, we intravenously injected RHAMM- and RHAMM+ HT29 and HCT116 cells into NSG mice. After 4 weeks, 8/8 mice injected with HCT116 and 4/5 mice injected with HT29 RHAMM+ cells developed multiple macroscopically detectable metastases in several organs, including liver, lung and kidney. Mice injected with RHAMM+ cells developed lymph node metastases in 5/8 (HCT116) and 4/5 cases (HT29) (Figure 4A, 4B). The metastatic nature of macroscopically detectable lesions was confirmed by histological evaluation (Figure 4B). Mice injected with RHAMM- HT29 cells did not develop any metastases (0/5), whereas only one mouse injected with RHAMM- HCT116 developed a metastasis (lung). Furthermore, after 4 weeks, EpCAM positive circulating tumor cells were found almost exclusively in mice injected with RHAMM+ cells (Figure 4C).

### RHAMM-positive CRC tumors express genes correlated with increased cell-cell and cell-surface detachment, motility and proliferation

**Figure 5:**
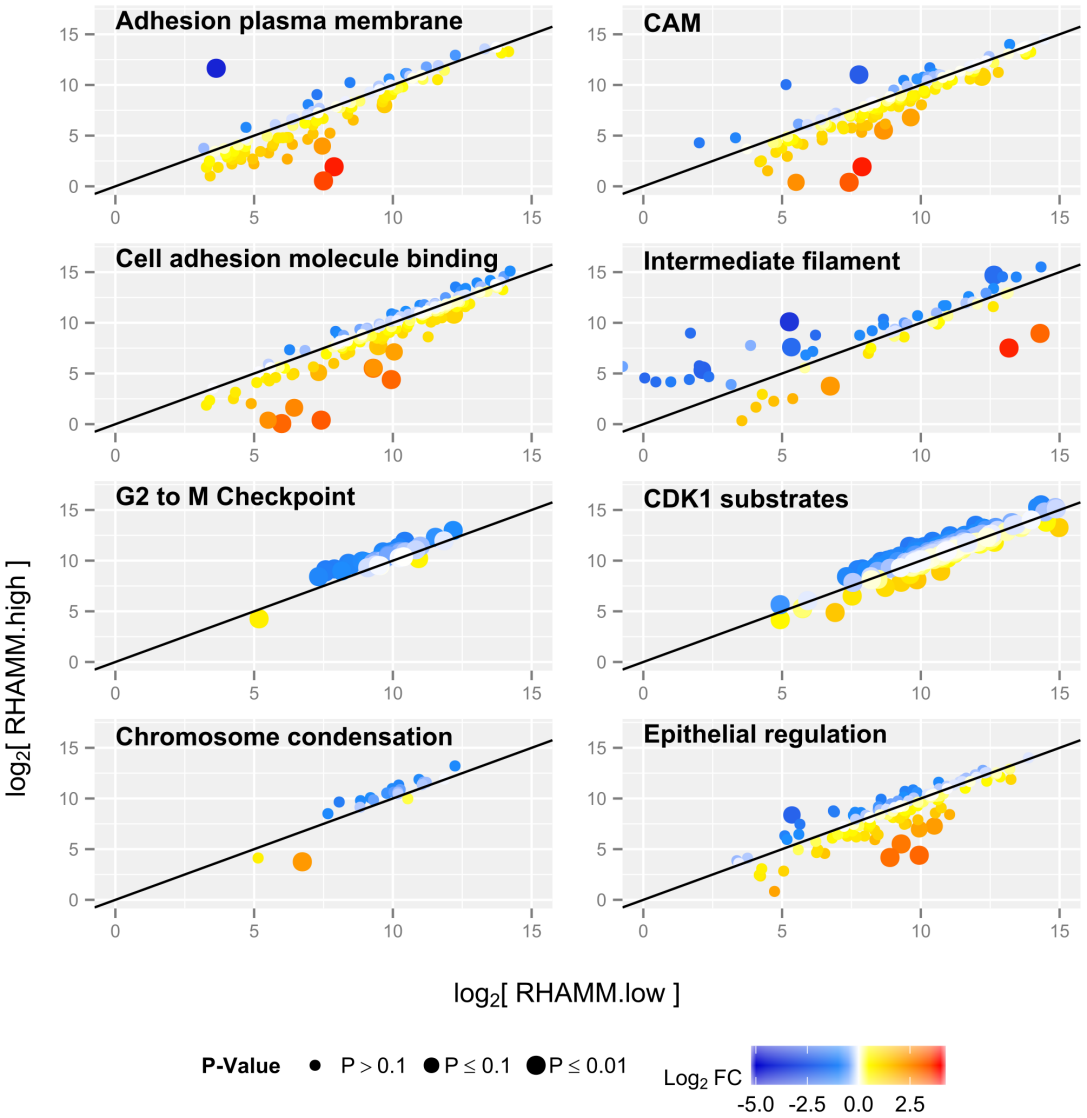
Pathway changes in RHAMM low versus RHAMM high human CRC samples Each dot represents one transcript, and values are shown as log2 fold changes. In RHAMM low tumors, mRNA coding for factors regulating cell-cell and cell-substrate binding are upregulated (yellow and red). In RHAMM high tumors, pathways involved in the cell cycle are upregulated (blue). P-value is expressed as dot size: the larger the dot, the more significant the P-value for differential expression between the two groups, adjusted for multiple testing.

RNA-Seq was performed on samples from stage-matched primary RHAMM high (n=3) and RHAMM low (n=3) CRC samples as determined by IHC. Proteins in pathways permitting cell cycle progression were upregulated in RHAMM high as compared with RHAMM low tumors. Conversely, we detected a downregulation in the expression of mRNAs coding for factors that regulate focal adhesion turnover, cell-substrate and cell-cell adhesion in RHAMM high tumors. These and other significantly altered pathways (Figure 5) and genes (Supplementary data) of interest are shown.

Increased proliferation of RHAMM high tumors which were selected for RNA-Seq was confirmed with Ki-67 immunohistological staining. All 3 RHAMM high tumors expressed Ki-67 in over 75% of the tumor cells, whereas the RHAMM low tumors showed an average of 40% of Ki-67 expression Interestingly, RHAMM low tumors also show lower levels of cleaved Casp3 (Supplementary Figure 5).

### RHAMM expression in matched primary, lymph node and distant metastasis

**Figure 6:**
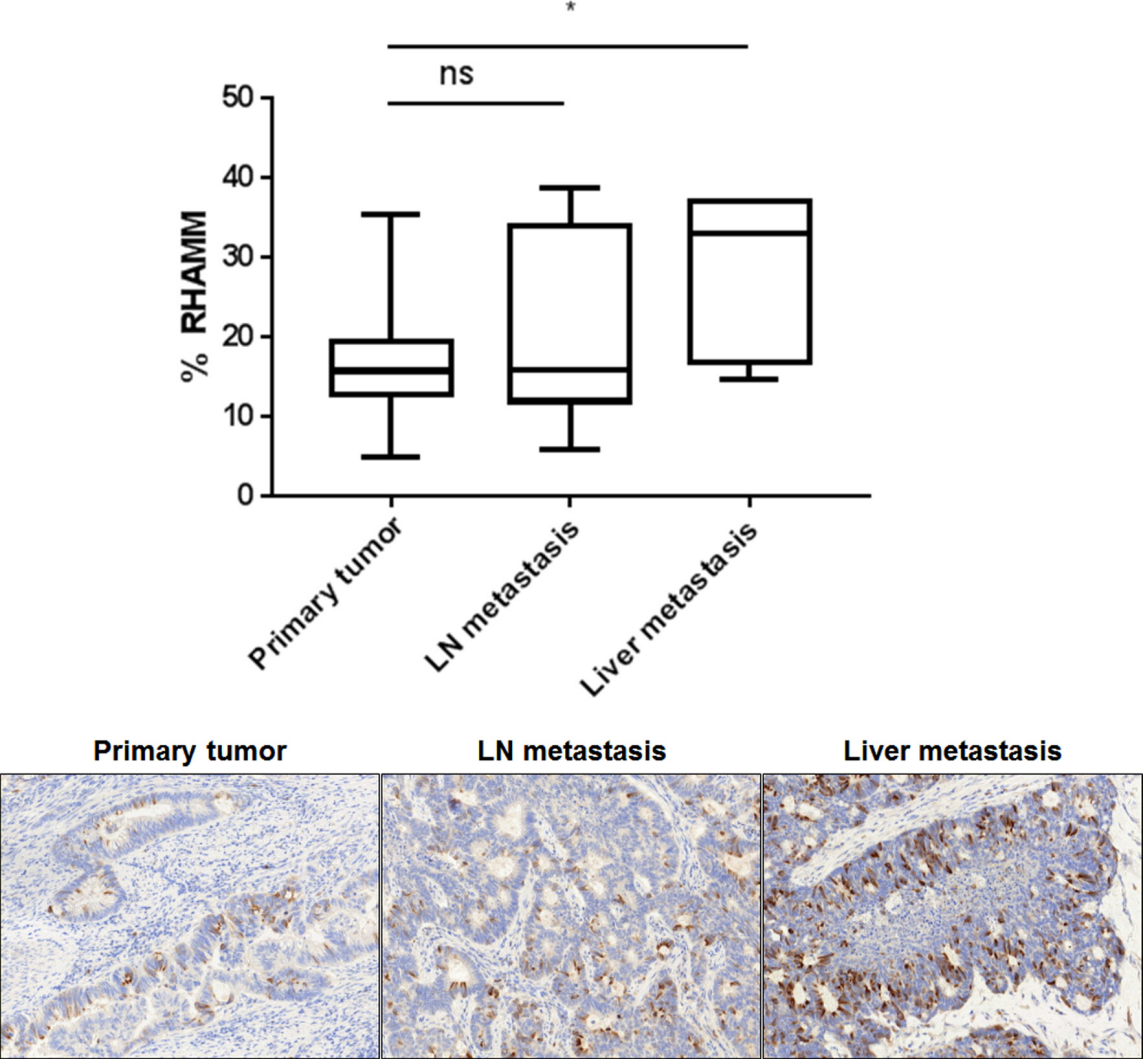
The percentage of RHAMM positive tumor cells increases from the primary tumor to the liver metastasis FFPE blocks from seven matched primary tumors, lymph nodes and liver metastasis were sectioned and immunohistochemically stained for RHAMM. Whole tissue slides were scored. Box whiskers show minimum and maximum values. The bar indicates the median value. Representative RHAMM IHC stains are shown.

The correlation of RHAMM expression with tumor cell spreading was evaluated on 7 matched samples from primary tumors, lymph node and liver metastasis. Notably, the percentage of RHAMM positive cells increased from primary tumors (mean 17%) to liver metastases (mean 37%, p=0.045, Figure 6), with the lymph node expression falling in between.

## DISCUSSION

In previous studies, we identified RHAMM overexpression as a marker of an aggressive phenotype and unfavorable clinical outcome in CRC [18–20]. However, whether RHAMM expression is a direct cause of tumor spreading remained unclear. Here, to test direct causation, we analyzed the effect of RHAMM knockdown on cell cycle, proliferation, migration, the invasion potential and tumorigenicity of CRC cells *in vitro* and *in vivo*. Moreover, to try and better understand the underlying causes of RHAMM effects on tumor cell behavior in CRC, we also evaluate the differences in various cellular pathways by testing genes expression in primary tumors discordant for RHAMM by RNA-Seq [22].

We originally described RHAMM as a potential prognostic factor in colorectal cancer patients [19]. In the present study we can confirm on a new, well characterized cohort, that patients with RHAMM high CRC tumors have a shorter survival time.

To select an appropriate cellular system, we measured RHAMM levels in 8 different, commonly used CRC cell lines. All showed high and comparable levels of intracellular RHAMM, and cell surface expression could additionally be induced by incubation with hyaluronic acid (HA), as previously described for other cell types [5, 6, 23]. We selected representative MMR positive (MMR+) HT29 and negative (MMR-) HCT116 cell lines for further assays. Interestingly, *in vitro*, both MMR+ and MMR- RHAMM- cells were less motile, had lower invasive capabilities, and displayed reduced proliferation rates and occupancy of S and G2/M cell cycle phases.

Notably, effects of RHAMM silencing on proliferation and cell cycle were only detectable in the presence of HA (data without HA not shown). As the interaction partner of RHAMM in the ECM, HA is present in different forms in quiescent and in inflamed tissues, including the tumor stroma, where it is degraded by reactive oxygen species and secreted enzymes to a much smaller, pro-inflammatory low molecular weight oligomer (LMWHA [3]). This degraded molecule can be bound to alternate combinations of receptors, and elicits different downstream responses within a cell [3]. Therefore, in the future it would be advantageous to establish a 3D co-culture setting to test the effect of RHAMM downregulation in the presence of HA of varying sizes.

RHAMM deficient cells were significantly less tumorigenic *in vivo*. Subcutaneous tumor formation was more rapid in mice injected with RHAMM+ compared with RHAMM- cells. The tumors likewise reached a much larger overall size. Unfortunately, this subcutaneous tumor model of CRC yields compact tumors with a largely predominant pushing border, in which we could not detect any tumor buds in either group, and could therefore not measure a change in bud numbers or infiltration depth. Nevertheless, RHAMM silencing virtually abolished the ability of HCT116 and HT29 cells injected into the tail vein to form metastases. While the majority of mice injected with RHAMM+ cells developed multiple metastases in the liver, lung, kidney and lymph nodes, only a single mouse injected with RHAMM- cells developed a metastasis, and only in a single organ. This suggests that RHAMM is not only necessary for the dissemination of the primary tumor by contributing to proliferation, migration and invasion, but for some stage in the colonization of the secondary site as well. Our data are in line with previous reports describing a similar capacity of RHAMM to promote migration, extravasation and metastasis [22] formation in non-small cell lung carcinoma [24], melanoma [25] and pancreatic cancer [26].

Consistent with data obtained with cell lines, RNA-Seq of primary tumors discordant for RHAMM revealed that its overexpression is associated with gene expression patterns characteristic for weaker cell-cell and cell-substrate attachment, higher cell motility and increased proliferation. However, further studies are warranted to elucidate the molecular mechanisms underlying potential direct or indirect interactions between RHAMM and the identified genes.

Taken together, our findings show for the first time that RHAMM has a strong, direct effect on proliferation, migration and infiltration of CRC cell lines and tumors derived thereof, and suggest that this protein might serve as a possible target in CRC.

There are previous reports of targeting RHAMM in humans. In acute myeloid leukemia, myelodysplastic syndrome, and multiple myeloma, RHAMM activates cellular and humoral immune responses, and was thus chosen as a target for an anti-RHAMM peptide vaccination approach [27], which had some success in initial clinical trials. In these diseases, however, the specificity of the approach was an issue, as RHAMM was only weakly expressed on leukemic stem cells. Nevertheless, targeting RHAMM in CRC could prove more promising, as normal colonic mucosa is only sporadically and weakly positive for RHAMM. Therefore, a similar strategy might be fairly specific for tumor cells.

We propose that targeting RHAMM could specifically inhibit the growth and dissemination of CRC by targeting the main tumor body as well as tumor buds and micrometastatic initiator cells.

## MATERIALS AND METHODS

### Patient selection for next generation tissue microarray (ngTMA) construction

A well-characterized cohort of 335 non-consecutive, unselected colon cancer patients treated at the Inselspital in Bern, Switzerland between 2002 and 2011 was retrospectively included in this study. Clinical data were obtained from patient records including patient age at diagnosis, gender, tumor location and size, pre- and post-operative therapy, and overall survival time. An experienced GI pathologist (AL) reviewed all histomorphological data of the surgical resections. The clinicopathological features of all patients are listed in Supplementary Table 1. The median survival time was 79 months (95% CI 68-114). The use of patient data has been approved by the local ethics committee, (Kantonale Ethikkomission Bern #KEK-BE 200/14).

### ngTMA construction

The ngTMA was constructed as described before [29]. Ninety-four patients were excluded from the original cohort based on pre-operative treatment (n=51) or insufficient material (n=43) remaining on the tissue block. The ngTMA included two 0.6 mm tissue punches (cores) each from tumor center, tumor front, tumor stroma (including buds) and normal colorectal mucosa of 241 patients. Additionally, each slide contained a set of punches derived from eight large, colorectal tumors with a homogenous histology, to serve as an inter-slide control for staining reproducibility.

### Immunohistochemistry (IHC)

IHC was performed as described before [19, 28]. The specificity of the stain was confirmed by comparison with an alternate antibody (Novus EPR4055). This marker was used to confirm the previously reported relationship [19, 20] between the level of RHAMM expression in the tumor and overall survival, defined as the period of time in months from the date of diagnosis to the date of death.

Ki-67 (M7240, Dako) and cleaved Caspase 3 (#9664, Cell signaling) IHC stains were performed on whole tissues sections analogously, with small alterations in the pretreatment (Citrate buffer, 30 minutes, at 95°C and 100°C, respectively), and incubation time (1 hours for both). The percentage of positive tumor cells was scored.

### Evaluation of immunohistochemistry using digital image analysis

For the scoring of RHAMM staining on the ngTMA, RHAMM expression was calculated by using the percentage of tumor cells showing any marker positivity. An ngTMA spot was scored only when it contained more than 100 tumor cells. Normal mucosa served as an internal control, and RHAMM was evaluated as overexpressed in tumors when it exceeded the percentage of signal found in normal tissue (5%). The scoring was done both by eye by a postdoctoral fellow focusing on RHAMM (LS), an experienced gastrointestinal pathologist (HD), blinded to clinical data, and by a digital quantification software (HALO, Indica Labs v1.94). HALO algorithm specifications: Image Zoom:0.25; Class List: Tumor; Classify Registered: Nuclear Stain:0.181,0.151,0.095; Positive Stain1: 0.164,0.286,0.433; Stain1 Localization: Cytoplasm; Minimum Tissue OD: 3.7e-002; Tissue Edge Thickness: 0.; Nuclear Contrast Threshold: 0.525; Minimum Nuclear OD: 9.5e-002; Minimum Nuclear Size: 9.8; Maximum Nuclear Size: 571.7; Minimum Nuclear Roundness: 0.; Nuclear Segmentation Aggressiveness: 0.385; Fill Nuclear Holes: 1; Maximum Cytoplasm Radius: 5.; Stain1 Min OD Weak: 7.6e-002; Stain1 Min OD Moderate: 0.123; Stain 1 Min OD Strong: 0.268.

The interobserver agreement of RHAMM immunohistochemistry scores was excellent between two observers (r=0.88) and between an observer and image analysis software (r= 0.8).

### Tumor cell culture

Established human CRC cell lines HCT116, HCT15, DLD-1, HT29, SW480 and SW620 were purchased from European Collection of Cell Culture (ECACC) a year before the start of the project. HT29, LoVo and CACO 2 were either a kind gift or already present in the facilities. All cells were used within 10 passages from initial thawing. In addition, HT29 and HCT116 were authenticated by DNA fingerprinting prior to its use, and found to be a full match of the HT29 and HCT116 reference profiles. HCT116, HCT15, DLD-1, LoVo and CACO-2 were maintained in RPMI 1640 supplemented with 10% fetal bovine serum (FBS), GlutaMAX-I, non-essential amino acids (NEAA), 100 mM sodium pyruvate, 10 mM HEPES (all from GIBCO). HT29 was maintained in McCoy’s 5A medium (Sigma) supplemented with 10% FBS and GlutaMAX-I. SW480 and SW620 were cultured in L-15 Medium (Leibovitz) (Sigma-Aldrich) supplemented with 10% FBS and GlutaMAX-I. Kanamycin sulfate (GIBCO) was included with all media. Cells were cultured at 37°C with 5% CO2. Absence of mycoplasma contamination in cultured cells was verified by PCR testing prior to investigation.

### Flow cytometry

The cell surface and intracellular expression of RHAMM in a panel of CRC cell lines was tested by flow cytometry using a RHAMM-specific monoclonal antibody (abcam clone 2D6, ab67003). To evaluate the intracellular expression, cells were fixed and permeabilized using the BD FACS™ Permeabilizing Solution 2 (#340973, BD Biosciences), following the manufacturer's protocol and then stained with anti-RHAMM antibody. Propidium iodide (PI, 0.5 μg/ml) was added to all samples prior to analysis to exclude dead cells. Samples were analyzed by a dual laser BD FACS Calibur flow cytometer (BD Biosciences), following exclusion of dead cells based on PI incorporation. Analysis was performed using FlowJo software (FlowJo LLC, Ashland, OR, USA).

### Short hairpin RNA (shRNA) silencing

Five different bacterial cultures (MISSION® shRNA Bacterial Glycerol Stock Nr: TRCN0000061553; TRCN0000061555; TRCN0000333645; TRCN0000333646; TRCN0000333647, annotated 1-5) were purchased from Sigma-Aldrich, and were amplified for isolation of shRNA plasmid DNA. Self-inactivating replication incompetent viral particles were produced in packaging HEK293T cells by transfection of the purified plasmid. Target HT29 and HCT116 cells were infected with lentiviruses containing the different plasmids and selected for Puromycin resistance. Cells infected with a lentivirus carrying a scrambled shRNA sequence were used as a negative control. The efficiency of silencing was then tested by western blot and the cells transfected with the plasmid inducing the highest reduction in protein expression were selected and used for the functional experiments (Plasmid number 1 in Supplementary Figure 4).

### Western blot analysis

Cells lysates were obtained by using 50 mM Tris pH 8.0, 150 mM NaCl, 1% NP-40, 0.1% SDS. Protein concentrations were determined by the Pierce BCA protein concentration assay according to the manufacturer’s instructions (Thermo Scientific, Waltham, MA, USA). Blots were probed with mouse anti-RHAMM (1:1000, clone 2D6, ab67003, abcam) and goat anti-β-actin (1:2000, Santa Cruz, Santa Cruz, CA, USA) antibodies. Secondary species-specific HRP-conjugated IgG (Santa Cruz) together with Pierce ECL (Thermo Fisher, Scientific Waltham, MA, USA) were used for detection of immunoreactive proteins. The cells with the highest reduction in protein expression were selected for further experiments.

### Proliferation assay and cell cycle analysis

Three-thousand RHAMM silenced and control HT29 and HCT116 cells were seeded in a 96-well plate in the presence of 100 μg/ml low molecular weight HA (Sigma, 40583) and analyzed every 24 hours for 6 days using the CYQUANT cell proliferation assay kit (Invitrogen, Carlsbad, CA, USA), according to the manufacturer’s protocol. Five well replicates were used per experiment.

To detect a change in cell cycle progression, RHAMM silenced and control HT29 and HCT116 cells were trypsinized, fixed with 1% paraformaldehyde for 15 minutes and stored in 70% ethanol at -20°C for at least one hour. The cells were then centrifuged and stained with PI/RNase staining buffer (BD Biosciences) for 15 minutes, according to the manufacturer’s protocol. Upon staining the DNA content was analyzed by flow cytometry on a BD LSRFortessa instrument and the cell cycle profile was assessed by using FlowJo Software (FlowJo LLC, Ashland, Oregon). Five well replicates were used per experiment. Three different experiments with two technical replicates were performed.

### Migration and invasion assay

HT29 and HCT116 tumor cells were suspended in serum-free medium, and seeded in the upper chambers of transwell plates onto uncoated or Matrigel-coated membranes (8 μm pore size, Corning). After 20 hours, the number of cells that had migrated into the lower chambers was quantified by CyQUANT Cell Proliferation Assay Kit (Invitrogen, Carlsbad, CA, USA). The migration capacity was indicated with the relative fluorescent units (RFU) of cells migrated through the membrane and the invasiveness was calculated according to the following formula: (mean of relative fluorescent units (RFU) of cells invaded through Matrigel-coated membranes / mean RFU of cells migrated through un-coated membranes).

### Analysis of tumorigenic and metastatic potential *in vivo*

*In vivo* experiments were approved by the Cantonal Veterinary Office Basel-Stadt, license number 2266. Female NSG (NOD.Cg-Prkdcscid Il2rgtm1Wjl/SzJ,) mice from Charles River Laboratories (Sulzfeld, Germany), were bred and maintained under specific pathogen-free conditions in the animal facility of the Department of Biomedicine, University of Basel. To assess the tumorigenicity, 1×10^5^ HT29 or HCT116 cells, infected with lentiviral particles carrying either a RHAMM shRNA or a scrambled control, were suspended in a 1:1 PBS and Matrigel (BD Biosciences) dilution and injected subcutaneously in the flank of 8 week old NSG mice. Each group consisted of 4 animals. Tumor formation was monitored twice weekly by palpation and caliper measurements. Mice were sacrificed when the tumors reached a maximum size of 10 mm. Tumor volumes (in mm^3^) were determined according to the formula (length x width^2^/2).

To examine metastasis formation of RHAMM silenced versus wild type HT29 or HCT116 cells, 10^5^ cells were resuspended in 100 μl PBS and injected into the tail vein of NSG mice. After 4 weeks, metastasis formation in organs of interest (lungs, livers, kidney, and lymph nodes) was assessed and confirmed by histological evaluation on hematoxylin and eosin stains. The slides were scanned with the Pannoramic slide scanner (3DHISTECH) at 20x. The peripheral blood of the mice was taken immediately after the sacrifice in order to evaluate the presence of circulating tumor cells (CTCs) in the blood. CTCs were detected by staining with an anti-human EpCAM antibody (BD Biosciences, Switzerland; clone EBA-1; #347200) on the BD Calibur cytometer. The number of CTCs was normalized to the volume of blood taken.

### Patient selection for RNA-Seq

Six stage 2 primary tumors with either low RHAMM levels or RHAMM overexpression were selected from 56 random, non-consecutive CRC cases treated by surgery between 2010 and 2013 at the Bern University Hospital, based on RHAMM protein detection by IHC and availability of fresh material at the Tumor Bank Bern. Information on patient gender, age at diagnosis, pT (primary tumor), pN (regional lymph node metastasis), as well as presence and location of distant metastasis was extracted from patient files in accordance with the UICC TNM classification 7th edition. Patient characteristics are provided in Supplementary Table 2. For RNA-Seq analysis, full tissue sections were cut from each tumor set and tumor tissue was scratched under visual control to minimize contamination by non-neoplastic tissue. RNA was isolated from 15 mg tissue using the Absolutely RNA Miniprep Kit (Ambion, 400800).

### RNA-Seq data analysis

Between 30 and 45 million read pairs (2×100 bp) were obtained per sample and the quality of the reads was assessed using fastqc v.0.10.1 (http://www.bioinformatics.babraham.ac.uk/projects/fastqc/). The reads were mapped to the human reference genome (ensembl, GRCh37.75) with Tophat v.2.0.13 [29]. We used htseq-count v.0.6.1 [30] to count the number of reads overlapping with each gene, as specified in the ensembl annotation (release 75). The Bioconductor package DESeq2 v. 1.6.3 [31] was used to test for differential gene expression between conditions. In total, we performed four different pairwise comparisons, two between expression levels within tumor types and two between tumor types within expression levels. The P-values were adjusted for multiple testing using the false discovery rate approach of Benjamini-Hochberg as implemented in DESeq2. SetRank [32] was used to identify gene sets enriched for differentially expressed genes. The tool collects gene sets from eight different databases (GO, ENCODE, Pathway Interaction Database, Reactome, BioCyc, KEGG, PhosphoSitePlus and WikiPathways), and performs an enrichment analysis that accounts for overlap between gene sets.

### Statistical analysis

For survival assessment using non-dichotomized data, Cox regression analyses were performed. Hazard ratios (HR) and 95% confidence intervals (CI) were used to determine the effect size. Differences in survival time were displayed using dichotomized data and standard Kaplan-Meier curves and tested using the log-rank test in univariate analysis. The time of survival was defined as the time of an event occurrence (death) or censored (patient lost to follow-up) relative to the date of operation.

For the functional *in vitro* and *in vivo* assays, statistical analyses were performed using 2-tailed Student’s T-test, one-way ANOVA, or the Mann-Whitney-U test as appropriate. Analyses were performed using SPSS Version 23 (IBM Corporation). P-values ≤0.05 were considered significant.

## SUPPLEMENTARY MATERIALS FIGURES AND TABLES





## Abbreviations

CRC: colorectal cancer
TNM: tumor-node-metastasis
MMR: mismatch repair
shRNA: short hairpin RNA
EpCAM: epithelial cell adhesion molecule
ngTMA: next generation tissue microarray
IHC: immunohistochemistry
