# Supplementary material for: The hyaluronan-mediated motility receptor RHAMM promotes growth, invasiveness and dissemination of colorectal cancer
Source: Oncotarget. 2017 Aug 3;8(41):70617–29. doi: 10.18632/oncotarget.19904 (PMC5642581; doi:10.18632/oncotarget.19904)
Supplement: Supplementary file 1 [file oncotarget-08-70617-s001.pdf]

The hyaluronan-mediated motility receptor RHAMM promotes growth, invasiveness and dissemination of colorectal cancer

SUPPLEMENTARY MATERIALS

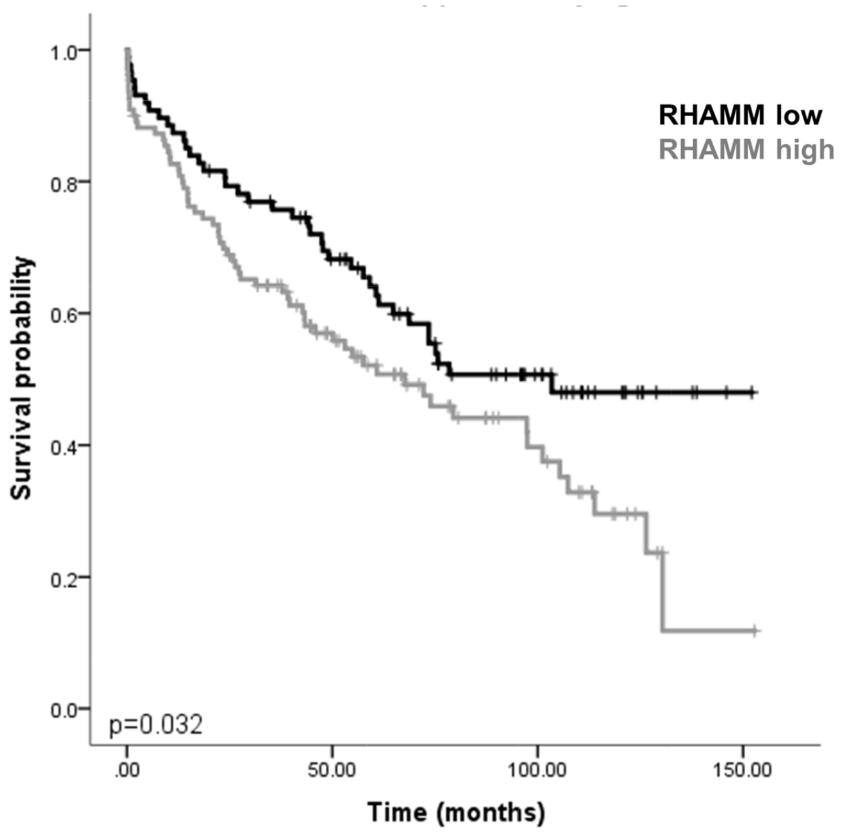

Supplementary Figure 1: Kaplan-Meier survival analysis of differential RHAMM expression. Patients with low RHAMM levels detected in the primary tumor have a higher 5-year survival rate (black: low RHAMM 0.64±0.04; gray: high RHAMM 0.49±0.05). Mean survival is 97±5, 95%CI 80-103 months for low RHAMM, 67±6 95%CI 55-79 months for high RHAMM expression.

A

Name	LoVo	DLD-1	HCT116	HCT15	SW620	SW480	HT29	CACO2
Growth	Adherent	Adherent	Adherent	Adherent	Adherent	Adherent	Adherent	Adherent
Morphology	Epithelial	Epithelial	Epithelial	Epithelial	Epithelial	Epithelial	Epithelial	Epithelial
Age	56	Adult	Adult		51	50	44	72
Gender	Male	Male	Male	Male	Male	Male	Female	Male
Localization	Colon: metastatic supraclavicular region	Colon	Colon	Colon	Colon: derived from metastatic lymph node	Colon	Colon	Colon
Microsatellite instability	instable	instable	instable	instable	stable	stable	stable	stable

B

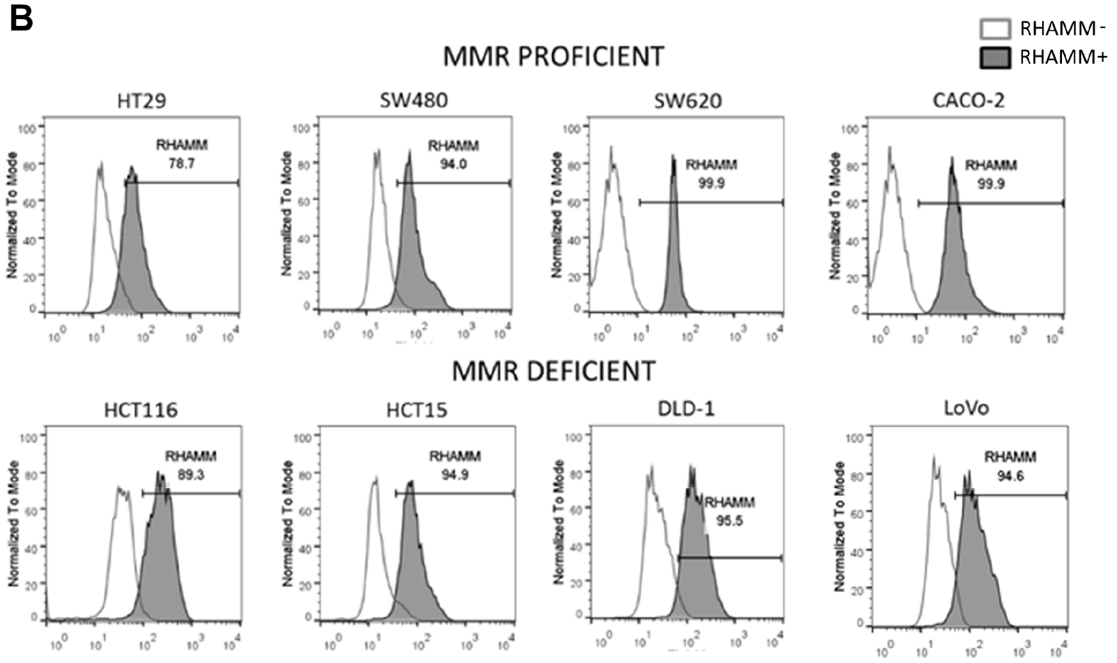

Supplementary Figure 2: **(A)** Characteristics of 8 established CRC cell lines used in this study. **(B)** RHAMM expression in CRC cell lines tested by flow cytometry. The percentage of RHAMM expression (shaded gray) is shown for each cell line.

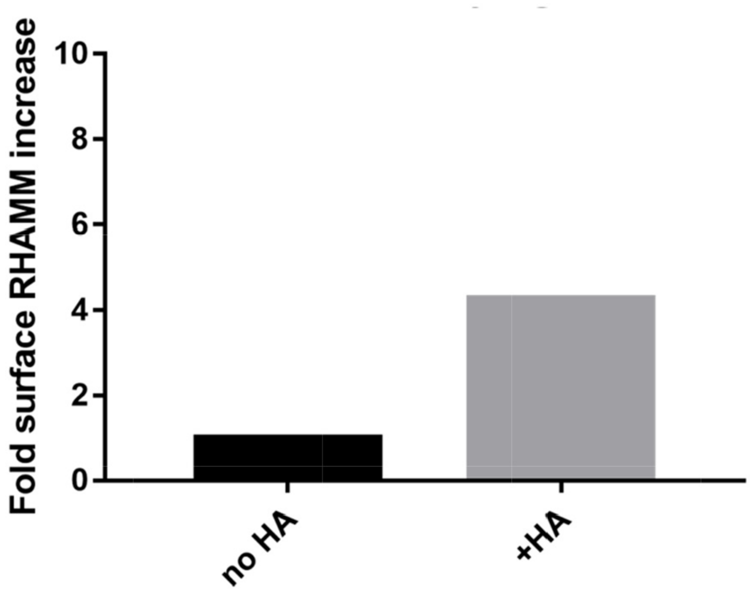

Supplementary Figure 3: RHAMM surface expression can be induced by the addition of hyaluronic acid. HT29 cells were seeded in medium containing 100 µg/ml hyaluronic acid at ~50% cell density. The cells were allowed to grow for 48 hours, then quickly trypsinized, washed and stained with anti-RHAMM primary antibody and an anti-rabbit fluorescently labeled secondary antibody. Untreated cells served as a negative control. RHAMM surface expression was analyzed by FACS.

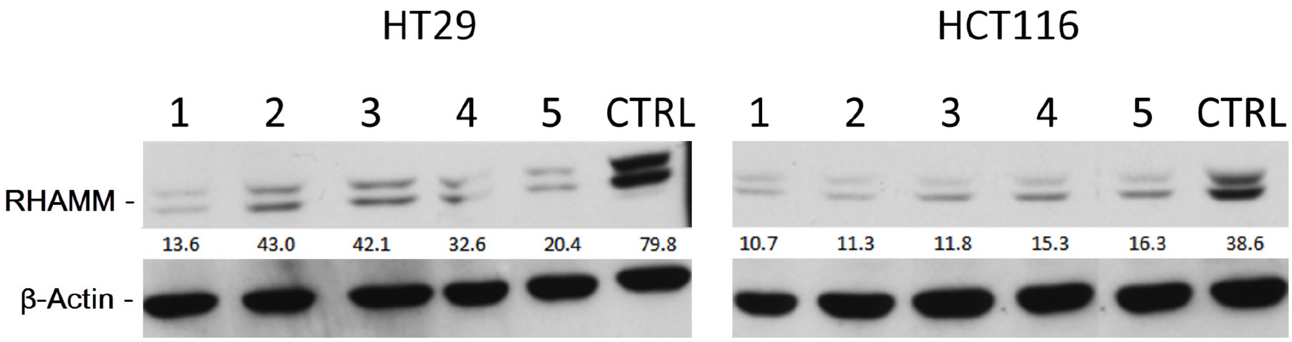

Supplementary Figure 4: Stable HT29 and HCT116 cell lines were generated by lentiviral transfection of a RHAMM-specific and control scrambled shRNA. Knockdown was confirmed by western blot. Upper panel: lanes 1-5 show RHAMM expression in cells transfected with five different RHAMM shRNAs. "CTRL" shows RHAMM expression in cells transfected with a scrambled control. Lower panel: Beta actin was used as a loading control.

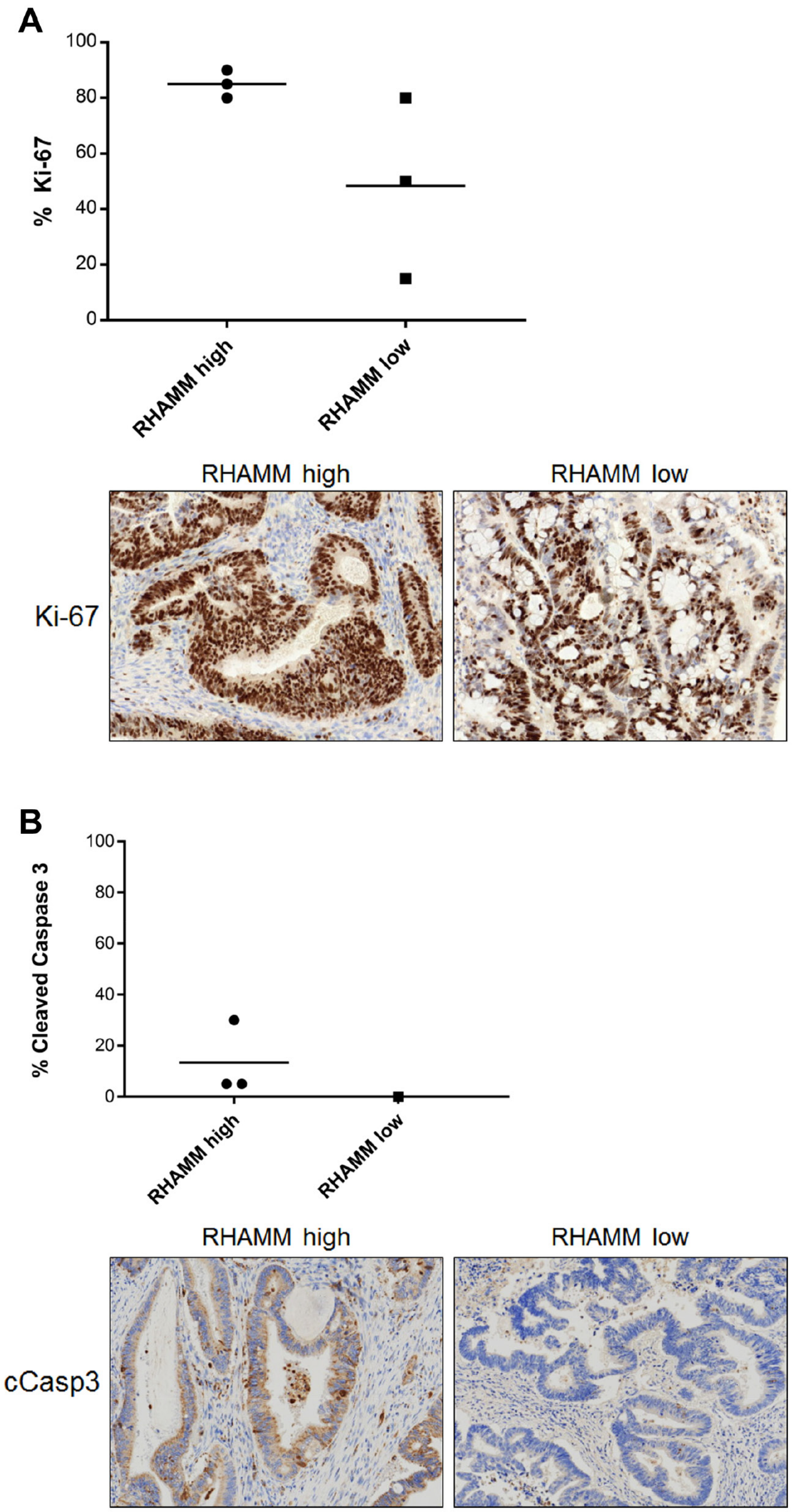

Supplementary Figure 5: Ki-67 and cleaved Caspase 3 levels are lower in RHAMM negative colorectal cancers. FFPE slides from three stage-matched cases of CRC were IHC stained for Ki-67. A: Ki-67 and B: cCasp3 expression in stage-matched cases chosen for RNA-Seq. Representative IHC stains are shown.

Supplementary Table 1: Distribution of clinicopathological features in the CRC patient cohort (n=241)

Features		Frequency N (%)
**Age (yrs) (n=241)**	Median (min, max)	71.5 (19.2, 91.5)
**Gender (n=241)**	Male	145 (60.2)
	Female	96 (39.8)
**Histological subtype (n=241)**	Mucinous	37 (15.4)
	Non-mucinous	204 (84.6)
**Tumor location (n=241)**	Left	103 (42.7)
	Rectum	90 (37.3)
	Right	48 (19.9)
**pT classification (n=241)**	pT1	1 (0.4)
	pT2	43 (17.8)
	pT3	132 (54.8)
	pT4	65 (27)
**pN classification (n=240)**	pN0	100 (41.5)
	pN1-2	140 (58.1)
**Positive lymph nodes (n=241)**	Median (min, max)	1 (0, 37)
**Metastasis (diagnosis) (n=241)**	M0	212 (88)
	M1	29 (12)
**TNM stage (n=241)**	I	25 (10.4)
	II	64 (26.6)
	III	123 (51)
	IV	29 (12)
**Tumor grade (n=236)**	G1-2	172 (71.4)
	G3	64 (26.6)
**V classification (n=225)**	V0	89 (36.9)
	V1-2	136 (56.4)
**L classification (n=221)**	L0	54 (22.4)
	L1-2	167 (69.3)
**Post-operative therapy (n=239)**	None	176 (73)
	Yes	63 (26.1)
**Survival (n=241)**	Alive/Censored	146 (60.6)
	Death	95 (39.4)
**Overall survival time (n=241)**	5-year (%)	57.1

Supplementary Table 2: Basic clinicopathological features in the CRC cases selected for RNA-Seq

RHAMM overexpressed	RHAMM %	pT	pN	Stage
1	70	3	0	2
2	80	3	0	2
3	80	3	0	2

RHAMM low	RHAMM %	pT	pN	Stage
4	20	3	0	2
5	10	3	0	2
6	20	3	0	2

Supplementary Data:

See Supplementary File 1
